# Trans-Arterial Stem Cell Injection (TASI): The Role of Interventional Radiology in Regenerative Medicine

**DOI:** 10.3390/jcm13030910

**Published:** 2024-02-05

**Authors:** Makoto Taninokuchi Tomassoni, Yinghui Zhou, Lorenzo Braccischi, Francesco Modestino, Junji Fukuda, Cristina Mosconi

**Affiliations:** 1 Department of Radiology, IRRCS Azienda Ospedaliero-Universitaria di Bologna, Via Albertoni 15, 40138 Bologna, Italy; lorenzo.braccischi@studio.unibo.it (L.B.);; 2 Faculty of Engineering, Yokohama National University, 79-5 Tokiwadai, Hodogaya-ku, Yokohama 240-8501, Kanagawa, Japanfukuda@ynu.ac.jp (J.F.)

**Keywords:** interventional radiology, stem cell therapy, regenerative medicine

## Abstract

Regenerative medicine is taking a step forward in treating multiple diseases. The possibility of renewing damaged tissues with stem cells has become a topic of interest in recent decades. Still a relatively new research topic, many issues in this discipline are being addressed, from cell culturing to the study of different graft materials, and, moreover, cell delivery. For instance, direct intravenous injection has a big downfall regarding its lack of precision and poorly targeted treatment. Trans-arterial and direct percutaneous infusion to the aimed tissue/organ are both considered ideal for reaching the desired region but require image guidance to be performed safely and precisely. In this context, interventional radiology becomes pivotal for providing different cell delivery possibilities in every case. In this review, we analyze different basic stem cell therapy concepts and the current and future role of interventional radiology with a focus on trans-arterial delivery.

## 1. Introduction

Stem cell therapy holds the promise of treating diseases that cannot be cured by conventional drugs, since tissues and organs are generated from stem cells in embryogenesis [1]. However, some studies have shown that stem cell therapy alone has limitations, which include delivery of cells to specific tissues, cell survival after transplantation, and control of long-term immune and inflammatory reactions [2]. To address these limitations, stem cell therapy has been combined with tissue engineering, genetic engineering, immunology, and developmental biology, leading to regenerative medicine [3,4,5]. The ultimate goal of regenerative medicine is to restore tissue structure, promote organ function, reduce disability, or improve quality of life [6]. Regenerative medicine is a promising approach to treating diseases that are incurable with traditional drug therapies and surgery, and its potential applications in a wide range of diseases have been extensively studied. Although significant progress has been made in regenerative medicine, the delivery of stem cells to the target tissues is still lagging [7]. Traditional delivery methods such as intravenous injections or infusions can result in a large percentage of the cells being lost or destroyed before they reach the target tissue, limiting their efficacy [8]. Interventional radiology (IR) techniques have emerged as a promising solution to this problem [9]. IR is a medical specialty that uses imaging tools such as X-rays, ultrasound, and MRI to guide minimally invasive procedures. These procedures are performed through small incisions or punctures in the skin and can often be performed on an outpatient basis with minimal discomfort and a shorter recovery time than traditional surgery [10].

The main benefits of IR in regenerative medicine can be summarized in terms of three aspects:

(a) Minimally invasive: Interventional radiology procedures are minimally invasive, reducing the risks associated with open surgeries and promoting faster recovery for patients.
(b) Precise and safe: Advanced imaging technologies allow interventional radiologists to visualize the delivery process in real time, ensuring accurate placement of stem cells and reducing the likelihood of complications.
(c) Repeatable and scalable: Interventional radiology procedures are repeatable, enabling multiple stem cell injections over time. This scalability allows for personalized treatment plans based on the patient’s response and disease progression.

One of the key advantages of IR in stem cell delivery is the ability to deliver cells directly to the target tissue. This is particularly important for conditions such as organ failure diseases, where the damaged tissue is often difficult to access. In these cases, stem cells can be delivered directly to the desired organ through a catheter inserted into the feeding artery. The catheter is guided to the target area using real-time fluoroscopy, and the stem cells are delivered precisely where they are needed [9].

The aim of our work is to review the current scientific evidence of intra-arterial fluoroscopic-guided delivery of stem cells, allowing us to take a step forward towards a safer and more precise stem cell therapy. In the future, trans-arterial stem cell injection (TASI) might become a pivotal procedure in the field of regenerative medicine.

## 2. Cell and Tissue Grafts for Regenerative Medicine

**Figure 1:**
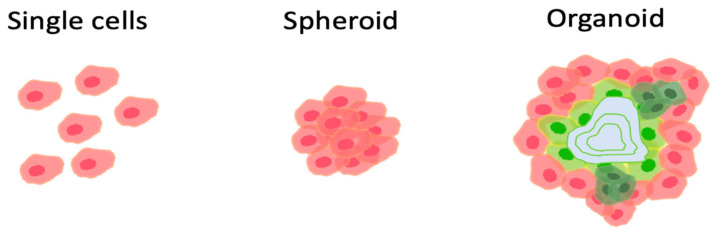
Modalities of tissue grafts. In regenerative medicine, tissue grafts are prepared in various forms, such as single cell suspensions, cell encapsulated hydrogels, spherical cell aggregates (spheroids), two-dimensional cell aggregates (cell sheets), and organoids.

In regenerative medicine, grafts are prepared in various forms, such as single cell suspensions, cell encapsulated hydrogels, spherical cell aggregates (spheroids), two-dimensional cell aggregates (cell sheets), and organoids (Figure 1) [11]. These forms largely affect the way in which cells are delivered to target tissues and organs. In addition, there are various routes of graft delivery, depending on the target tissues and organs, such as direct injection, delivery through blood vessels, adhesion to the tissue surface, and replacement of the entire tissue. The appropriate combination of a tissue form and a delivery approach is vital to minimize invasiveness and maximize therapeutic efficacy.

### 2.1. Single Cells

Direct intravascular injection of stem cell suspension has been used to treat damaged tissues and organs. One of the most advanced examples of this approach is the treatment with mesenchymal stem cells (MSCs). MSCs are a type of cells with self-renewal capacity, multispectral potential, and immunosuppressive properties, and work in the spontaneous regeneration process of several tissues and organs [12]. When trauma occurs, MSCs prompt cell renewal and migrate to damaged tissue for regeneration [13]. The ability of MSCs to proliferate and differentiate into various cell types in vitro makes them a very promising tool for the treatment of organ failure, chronic wounds, dental regeneration, and neurodegenerative disorders such as Alzheimer’s disease, Parkinson’s disease, and stroke, as well as many other diseases [1]. The challenge in direct injection of stem cells is caused by the fact that adherent cells are intrinsically programmed to undergo apoptosis in suspension. Hydrogels, microcarriers, and porous scaffolds have been used to improve the cell viability after injection [14]. Hydrogels are a class of materials suitable for tissue engineering and drug delivery because of their ability to absorb large amounts of liquid, excellent biocompatibility, and outstanding diffusion properties [15]. Microcarriers are usually particles with a diameter of a few hundred micrometers, prepared from polysaccharides, proteins, or polymers, and are widely used in tissue engineering as carriers for cells, drugs, and growth factors [16]. Porous scaffolds can play an important role as a temporary support to accommodate seeded cells, to control their function and to guide the regeneration of tissues or organs, and are mostly prepared from biodegradable polymers and calcium phosphate [17]. These are typically designed to be biocompatible and biodegradable, and provide temporal mechanical and physical support [18]. Stem cells encapsulated in micro-hydrogels can be expanded, differentiated, and injected intravascularly, subcutaneously, or intraperitoneally [18].

### 2.2. Spheroids

Another approach for the improvement of the cell viability after injection is to form three-dimensional cell aggregates such as spheroids [19]. Cells in spheroids maintain their survival by inhibiting apoptosis through cell–cell adhesion. Spheroids are sometimes called embryoid bodies for stem cells and neuro spheres for neural stem cells. A spheroid culture provides a better environment for maintaining stemness and differentiation potential compared to a conventional two-dimensional culture [20]. MSCs in a spheroid culture possess enhanced anti-inflammatory, angiogenic, anti-fibrotic, and tissue-repairing effects [21]. Embryonic stem cells (ESCs) were differentiated into hepatocyte-like cells in a spheroid culture, which showed morphological and ultrastructural features of hepatocytes, and expressed significantly greater liver-specific genes and proteins than those in a conventional monolayer culture [22]. Injection of pancreatic β cell spheroids was found to be an effective treatment for type 1 diabetes in animal experiments [23]. Although spheroid injection faces many of the same challenges as cell suspension injection [24], spheroid-based cell delivery is a more effective strategy in regenerative medicine for various tissues and organs [25]. Currently, there are no studies using intra-arterial spheroid injections in humans, regardless of their demonstrated potential for stroke in an animal model using intra-arterial injection [26].

### 2.3. Cell Sheets

Two-dimensionally connected cell sheets have been used to replace damaged tissue surfaces or provide biological cues to promote intrinsic tissue regeneration [27]. Since tissues and organs are typically composed of various boundaries, transplantation of cell sheets to tissue surfaces, such as the cornea and gastrointestinal tract, would be a reasonable approach. Clinical research has shown the feasibility of cell sheet technology for the treatment of several tissues and organs (e.g., heart, liver, cornea, and bone) [28]. Although cell sheets are unsuitable for intravascular injection, endoscopic and minimally invasive transplantation to a specific tissue location is applicable. Endoscopically guided cell sheet delivery was successfully achieved to transplant human mesenchymal stem cell (MSC) sheets into the anterior and lateral walls of the left ventricle [29]. This approach would provide an alternative cell delivery and treatment approach for tissues and organs to which it is difficult to deliver cells using intravascular injection.

### 2.4. Organoids

Recent remarkable advances in stem cell research made it possible to induce various organoids in vitro, including the brain [30], liver [31,32], pancreas [32], stomach [33] lungs [34], and mammary glands [35]. Because organoids replicate features of specific organs, including vasculatures in some cases, more efficient engraftment, survival, and therapeutic effects are expected using an intravenous injection compared to typical spheroids. For example, vascularized liver organoids became functional by connecting to the host vessels within 48 h, and mesenteric transplantation of the organoids rescued lethal liver-failure animals [31]. Organoids with vasculatures that can be surgically anastomosed with host vessels have not yet been formed, but the degree of replication of tissue structures, including vasculatures, as well as the dimensions of organoids, should be closely associated with the selection of cell delivery to the target organ.

### 2.5. Extracellular Vesicles

In regenerative medicine, in addition to the stem cells themselves, the products secreted by stem cells have gained attention in recent years. Extracellular vesicles (EVs) are small endogenous vesicles released by various types of cells and are found in large quantities in body fluids. Once considered a cellular garbage bin [36], EVs are now in the spotlight because they exchange biological information between cells and participate in a variety of physiological and pathological processes. Recent studies have shown that EVs are involved in the regulation of apoptosis, cell proliferation, differentiation, migration, angiogenesis, oxidative stress, aging, and inflammation [37,38]. It has been shown that proteins, mRNAs and micro RNAs (miRNAs) in EV composition are involved in mediating tissue repair and regeneration [38].

The application of EVs as disease treatments or tissue repair agents can be categorized into two types: therapies that utilize the natural biological functions of EVs to mimic the natural repair process, and drug delivery methods that use EVs as carriers to deliver the therapeutic entity to the repair site. Studies on MSC-derived EVs have shown that their biological activity is comparable to that of parental cells, and their clinical applications are promising [39]. Since EVs are not living organisms, they pose less risk of tumorigenicity than parental cells [40] and reduce the safety risk regarding live stem cell injections [41]. In addition, EVs are considered good natural drug delivery systems and can be used for injectable therapies in regenerative medicine due to their low immunogenicity and bilayer membrane structure, which binds drug molecules (Table 1) [42].

## 3. Treatment of Specific Diseases with TASI

**Figure 2:**
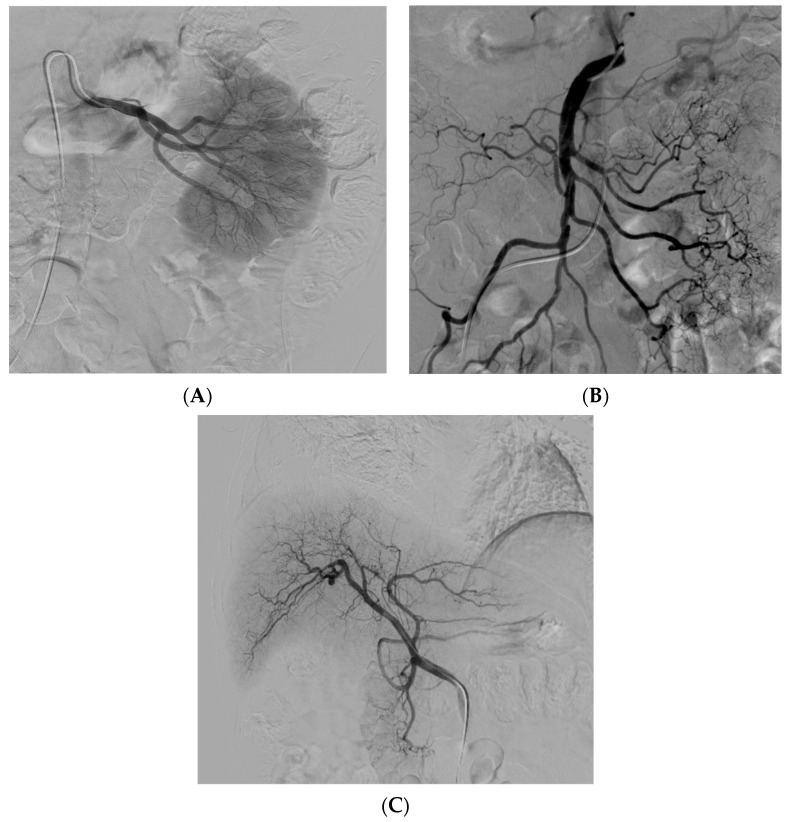
Angiographic catheterization of the renal (**A**), superior mesenteric (**B**), and hepatic artery (**C**). The catheter positioning ensures selective treatment into the desired tissue, avoiding non-target stem cell infusion.

Many issues still need to be addressed in the field of stem cell therapy, one of which is to determine the ideal cell delivery method. Although some studies suggest that direct intravenous stem cell injection might have some level of effectiveness, it has been demonstrated that this could also lead to lung entrapment and therefore reduce stem cell perfusion to the target tissue [7]. TASI might be considered an ideal selective delivery method with an optimal distribution and a high stem cell concentration in the desired organ. After tissue sampling and processing for stem cell isolation (autologous or heterologous), the procedure basically consists of catheter introduction through arterial access (most commonly femoral). Then, using fluoroscopic and contrast media guidance, the catheter is placed in the desired feeding artery (hepatic, mesenteric, or renal artery) for the subsequent stem cell injection (Figure 2).

Animal studies have demonstrated that TASI is a safe and effective treatment for multiple diseases, yielding solid preclinical evidence to encourage clinical studies [43,44,45,46,47]. Human studies have also indicated that TASI could be useful for managing multiple diseases, from parenchymal organs to osteoarticular conditions.

### 3.1. Liver Disease

Chronic liver disease is a severe medical condition that poses significant challenges to patients and healthcare providers alike. Liver transplantation is currently the most effective treatment for end-stage liver failure. However, it is hampered by the scarcity of donor organs and the risk of organ rejection, which requires lifelong immunosuppressive therapy [48]. Additionally, not all patients are suitable candidates for transplantation, making the search for alternative therapies crucial.

Chronic liver disease has recently been considered a manageable condition through TASI. Many studies suggest that the infusion of MSCs is a valid option for treating liver cirrhosis. Sakai et al. [49] conducted a multi-institutional clinical trial involving seven patients with non-alcoholic steatohepatitis (NASH) or fatty-liver-disease-related cirrhosis. The study consisted of fat extraction from the subcutaneous tissue of the patient’s abdomen or buttocks, isolation of autologous adipose-tissue-derived MSCs, and, subsequently, TASI. The patients were followed for 24 weeks through laboratory exams (serum albumin concentration and prothrombin activity). After 3 months, six out of seven patients’ serum albumin concentration improved and five out of seven patients’ prothrombin activity improved. After 24 weeks, all patients were Child–Pugh A. In another clinical trial, Suk et al. [50] evaluated TASI using, in this case, autologous bone-marrow-derived mesenchymal stem cells (BM-MSCs) extracted from the posterior iliac crest in patients with alcohol-related cirrhosis. In this case, patients were separated into three groups: the first was treated with only one injection, the second with two injections (one after 1 month and the second after 2 months), and the third was control group. A total of 50 patients were enrolled and followed for 12 months, and were evaluated with pre-trial and post-treatment liver biopsies with fibrous quantification (after 6 months). It was found that one-time and two-time TASI groups were associated with 25% (19.49 ± 9.48% vs. 14.51 ± 7.05%) and 37% (21.05 ± 8.94% vs. 13.22 ± 6.70%) reductions in the collagen proportion area (*p* < 0.001) compared to the control. Regarding clinical scores, Child–Pugh significantly improved in the one-time BM-MSC group (7.6 ± 1.0 vs. 6.3 ± 1.3; *p* = 0.035) and two-time BM-MSC group (7.8 ± 1.2 vs. 6.8 ± 1.6; *p* = 0.003). Conversely, the MELD score did not show a significant improvement compared to the control group. Finally, there was no evidence of BM-MSC-related tumors during the follow-up period.

A systematic review and meta-analysis of clinical trials using MSCs for chronic liver disease concluded that stem cell therapy is safe and effective in patients with chronic liver disease and TASI is considered the optimal delivery approach compared to intravenous infusion [51].

Although the only cure for liver cirrhosis is currently transplantation, TASI could be used as a bridge for patients waiting for transplantation and to prolong life expectancy.

### 3.2. Renal Disease

Renal failure is a serious disease that affects millions of individuals worldwide. Conventional treatments such as dialysis and kidney transplantation have provided significant relief to patients with this condition, but they are not without limitations.

Dialysis, a commonly employed treatment for renal failure, helps artificially filter blood. However, it is a temporary measure that requires regular sessions and is associated with significant lifestyle restrictions. Kidney transplantation offers a more permanent solution, but it is limited by the scarcity of donor organs and the risk of rejection.

It has been shown in multiple animal studies that stem cell therapy could also be beneficial in this clinical scenario [46].

In a clinical study [52], extracellular vesicles derived from umbilical cord mesenchymal cells injected directly into the kidneys with a catheter placed in the renal artery were proven to be safe and to reduce immune reaction, urea, and creatinine levels in patients with chronic renal failure. A total of 40 patients were enrolled and, in this case, contemporaneous intravenous and intra-arterial injections of EVs were performed.

In another scenario, TASI of autologous BM-MSCs was performed in 11 patients. The treatment in this case was also complemented with two intravenous injections. The patients were followed for one year, and at the end of the study an improved renal function was documented in six patients (54.5%). Conversely, a worsening was found in two patients (18.2%) [53].

Renovascular disease could also be treated with TASI. Saad et al. [54] developed a clinical trial with 14 patients treated with a single infusion of MSCs associated with standard medical treatment. After three months, an increase in cortical perfusion and renal blood flow was found. In this study, TASI was proven to be safe and able to ameliorate renal blood flow in patients with renovascular disease.

### 3.3. Diabetes

Type 1 diabetes mellitus (T1DM) is a chronic autoimmune disease that typically presents in childhood or early adulthood [55]. The most common treatment for TD1M is lifelong insulin replacement therapy. TASI has been proposed as a valid option to replace pancreatic function.

A recent systematic review and meta-analysis conducted by Madani et al. [56] analyzed 27 studies focusing on changes in the insulin total daily dose (TDD) level as the primary outcome and the changes in the glycated hemoglobin (HbA1c), c-peptide, and adjusted HbA1c levels as secondary outcomes. They divided studies into three groups: the transplantation of either only MSCs or hematopoietic stem cells (HSCs), or the combination of both. They included targeted and untargeted transplantations. Only a few authors have used trans-arterial access and X-ray guidance to deliver stem cells to the target. In fact, most diabetic patients undergo MSC or HSC transplantation through peripheral vein injection, while only a few patients receive MSCs or HSCs via pancreatic circulation. Considering patients who undergo a combination of HSCs and MSCs, one trial performed an injection of HSCs via peripheral blood and an injection of MSCs via the dorsal pancreatic artery. This trial, conducted by Cai et al. [57], confirmed that this route is a safe approach to help prevent the first-pass pulmonary impact of stem cells, minimizing their sequestration in the lungs and enabling the best outcomes. In other trials, the portal system was used most of the time. HSCs alone have shown poor results in terms of efficacy and significant side-effects in terms of opportunistic infections. For this reason, the meta-analysis does not suggest their use for TD1M therapy. Instead, the injection of MSCs alone is a safe treatment and has good results in terms of efficacy. Indeed, an insulin-free period (from 1 to 24 months) was observed in 10% of patients, while in 46% of patients a significant reduction in insulin TDD was reported. Nevertheless, the meta-analysis proved that the combination of MSCs and HSCs has shown better promise than the use of MSCs or HSCs alone. Their co-transplantation is a safe treatment for TD1M, with no important side effects and leading to a significant improvement of the disease. In fact, the insulin-free period was not observed, but more than 80% of patients experienced a reduction in insulin TDD. In addition, improvements in the HbA1c, c-peptide, and adjusted HbA1c levels were reported at 1 month follow-up, with a growing trend from 3 to 12 months. Despite these findings, the results in terms of efficacy are inconclusive due to the high level of heterogeneity, the lack of a control group, the absence of a complete follow up, and the lack of some crucial parameters for evaluating outcomes. In addition, most of the studies were conducted in dispersed institutes, without any consistency in the design of the studies, since the administration of stem cells in TD1M has not been approved by regulatory agencies. The authors suggest improving the quality of studies and standardization of outcomes [56].

### 3.4. Osteonecrosis

The osteonecrosis of the femoral head (ONFH) is a progressive condition resulting from a compromised blood supply to the affected femoral bone, leading to tissue death, secondary symptomatic hip arthritis, and eventual joint collapse, and requires total hip arthroplasty (THA) [58]. The early treatment guidelines for ONFH are still uncertain, and the precise indications have not yet been determined [59]. Although still in the early stages of investigation, clinical studies have shown promising results and have proved the safety of TASI for ONFH.

In 2017, Andriolo et al. [60] published an interesting meta-analysis and review including all of the regenerative techniques used for ONFH in order to compare their efficacy in combination with core decompression (CD) versus CD alone for reducing disease progression and conversion in THA. The authors divided all the studies into four groups considering different types of techniques. The second group included four papers about TASI. Firstly, a general analysis of cumulative survivorship showed optimal outcomes for the combination approach (80% of overall survival at ten years). Secondly, the authors conducted a stronger meta-analysis with only one level of RCT, including one of the TASI trials [61], to compare biological therapies with a control group. The analysis confirmed good results of all techniques in terms of femoral head survivorship compared to CD alone. Indeed, they found a total estimated cumulative survivorship of 95.6 for combination vs. 85.7% for CD alone at 24 months of follow-up, and 89.9 vs. 70.6% at 60 months of follow-up. This review provides solid evidence that all biological techniques, including TASI, combined with CD, could be expected to have a long-term effect on preventing ONFH patients from failure and THA [60].

A recent retrospective cohort study by Pan et al. [62] involving 35 patients (47 hips) in the early stages who underwent TASI demonstrated that the efficacy of autologous peripheral blood stem cells (auto-PBSCs) injection through arterial vessels is influenced by important pre-treatment factors. The primary endpoint was to avoid hip preservation failure, which means THA or other surgeries. They preferred using auto-PBSCs instead of MSCs due to the difficulty and high cost of collecting them. Firstly, they demonstrated that trans-arterial injection of auto-PBSCs is a safe and effective therapy for ONFH patients. Indeed, they found no complications and they reported a 50 ± 7% success rate of preserving the hip from surgery at 5 years follow-up, with a median overall survival of 60.18 months. Secondly, they showed that body mass index (BMI), Harris Hip score (HHS), age, and pre-treatment necrotic volume independently influence the efficacy of auto-PBSC injection. They finally proposed a nomogram model (C-index = 0.8863; score test *p* = 0.000) to predict hip preservation probability, including BMI (*p* = 0.012), HHS (*p* = 0.022), age (*p* = 0.042), and pre-treatment size of the lesion (*p* = 0.000). In fact, low age, necrotic volume, and BMI, and a high HHS, are associated with favorable outcomes.

Clinical papers involving only intra-arterial injection are few in number. Nevertheless, they have shown encouraging results, indicating potential benefits in terms of pain relief, functional improvement, and disease progression. Large-scale, well-designed randomized controlled trials are necessary to definitively establish the therapy’s efficacy. With continued investigation, stem cell therapy has the potential to revolutionize the management of osteonecrosis and provide a viable treatment option for patients.

### 3.5. Peripheral Arterial Disease

Atherosclerosis is the main cause of peripheral arterial disease (PAD), and in more than 10% of cases, PAD can lead to important complications such as critical limb ischemia (CLI). CLI is clinically individuated by chronic ischemic rest pain and peripheral ischemic lesions as ulcers or gangrenes, and it has a high rate of amputations and mortality after amputation [63].

The current therapy provides revascularization of the extremities via endovascular or surgical approaches. Nevertheless, in almost half of cases, vascular revascularization is not suitable; thus, these patients undergo amputations of the limbs [64]. Over the years, several trials and meta-analyses were conducted about the use of stem cells as an alternative therapy in “no-option” patients.

In 2016, Rigato et al. [65] published a systematic review and meta-analysis, including 19 RCTs, 7 nonrandomized trials, and 41 non-controlled trials, and considering amputation as the primary endpoint. Evaluating only RCTs, they found that the trials using only TASI (*n* = 3) were significantly fewer than those using intramuscular injection (*n* = 15), while both alternatives were used in just 1 study. Additionally, analyzing only delivery routes, they proved that only intramuscular administration was associated with a significant improvement in primary endpoints such as the amputation rate. The authors hypothesize that the presence of significant stenosis of leg arteries could be the cause of the reduced efficacy of TASI. In fact, stenosis would diminish the quantity of stem cells injected arterially. On the contrary, a direct comparative study by Klepanec et al. [66], including 41 patients, showed no differences between the two groups.

The meta-analysis of Rigato et al. suggests that stem cell injection for CLI is a safe treatment without any severe complications, but considering only placebo-controlled RCTs and RCTs, it has no significant efficacy effect on primary endpoints, including all delivery routes [65].

A more recent and complete systematic review and meta-analysis [67] reported 27 RCTs involving 1186 patients, suggesting that, for patients not treatable by revascularization, the injection of autologous stem cells is a safe and effective option. The analysis included eight variants of stem cells and only two different delivery routes. In fact, TASI was performed in only three studies and the intramuscular approach was used in the others. Due to the lack of data and the high risk of bias, the authors did not investigate which type of stem cell is most efficacious and which kind of route is preferable. Firstly, in eight RCTs (in which one performed TASI) side-effects were reported and a major complication happened in just one patient, i.e., a case of sepsis through the site of intramuscular injection. Secondly, Gao et al. [67] evaluated primary endpoints including amputation rate and ulcer healing rate (UHR), and secondary endpoints, consisting of ankle Brachial index (ABI), transcutaneous oxygen tension (TcO2), pain-free walking distance (PFWD), and rest pain score. Both primary and secondary endpoints improved after stem cell injections compared with conventional therapy. Indeed, the analysis showed PFWD, ABI, UHR, and TcO2 were higher, and amputation rate and rest pain score were lower, than in the control group. Despite these results, the analysis demonstrated that stem cell injection did not statistically significantly increase the extremities salvage rate (*p* = 0.64). Finally, they created a subgroup of patients with DM in order to evaluate the efficacy of stem cells, but none of the TASI studies was included in this sub-analysis.

Several studies and good meta-analyses were conducted on the efficacy of stem cells for CLI. Nevertheless, studies using the endovascular approach are far fewer than those using intramuscular injection. The current literature demonstrates the safety and temporary efficacy of this therapy for no-option patients, but further trials are necessary to evaluate which types of stem cells are more valid and their efficacy on long-term endpoints, such as limb salvage.

## 4. Challenges and Future Perspectives

The future of TASI holds promise in revolutionizing the treatment landscape for chronic diseases. However, as we navigate toward realizing these futuristic possibilities, significant challenges persist.

Firstly, precise control over the fate and behavior of delivered stem cells, ensuring their proper integration into the host tissue, and addressing potential immune responses are current hurdles. Stem cell therapies in clinical trials involve stem cells that need to be highly purified and need to meet clinical-grade standards prior to injection. The main risk in the use of embryonic stem cells, for example, is that teratomas originate from residual pluripotent cells that retain a potentially uncontrolled proliferative state. Therefore, purification steps and quality control deserve attention to ensure that differentiated progeny for clinical use have been effectively removed from teratoma-forming cells [68]. In the future, the safety of stem cell therapies needs further attention. The pre-infusion preparation of stem cells needs to ensure that there is no evidence of contamination by any viruses or exogenous factors and that the genetic stability of the stem cells is maintained [68]. The safety and the immune responses induced during and after treatment with highly purified stem cells should be taken into account, and potential long-term complications should be a concern.

Secondly, optimizing the scalability and reproducibility of TASI protocols is vital for clinical translation; stronger clinical trials on a multicenter basis are still needed to acquire more evidence of this procedure. Overcoming these challenges will be pivotal in unlocking the full potential of TASI, ushering in a new era of personalized and effective treatments for chronic diseases.

## 5. Conclusions

Regenerative medicine, empowered by stem cell therapy and interventional radiology, offers hope for patients with conditions such as liver failure and other disorders requiring tissue repair and regeneration. Interventional radiologists possess specialized skills in image-guided procedures, enabling precise placement of stem cells during trans-arterial injection. Their expertise in navigating the complex vascular anatomy and utilizing advanced imaging technologies ensures accurate delivery to the desired location. This collaboration between these disciplines paves the way for innovative and effective regenerative treatments. While the use of TASI shows promise, several challenges remain. Refining the selection and sourcing of appropriate stem cells, optimizing injection techniques, ensuring long-term safety, and conducting comprehensive clinical trials are vital for establishing the therapy’s efficacy and safety. Although there is still a long way to go, we believe through continued research and collaboration, we can unlock the full potential of image-guided therapy, ushering in a new era of regenerative medicine for the benefit of patients worldwide.

## Figures and Tables

**Table 1:** Summary of different cell culturing methods and their potential in the field of TASI.

Culture Type	Image Guided Cell Delivery Method	TASI Animal Studies	TASI Clinical Studies	Pathologies Treated with TASI in Clinical Studies
Single cells	- Intravascular and percutaneous	Yes	Yes	- Liver and kidney failure - Diabetes - PAD - Osteonecrosis
Spheroids	- Intravascular and percutaneous	Not performed yet	Not performed yet	-
Cell sheets	- Only percutaneous possible	-	-	-
Organoids	- Ideally percutaneous - Vascular possible	Not performed yet	Not performed yet	-
Extracellular vesicles	- Intravascular and Percutaneous	Yes	Yes	- Renal failure

## Data Availability

No new data were created or analyzed in this study. Data sharing is not applicable to this article.
